# Neurophysiological Markers of Premotor–Motor Network Plasticity Predict Motor Performance in Young and Older Adults

**DOI:** 10.3390/biomedicines11051464

**Published:** 2023-05-17

**Authors:** Sonia Turrini, Naomi Bevacqua, Antonio Cataneo, Emilio Chiappini, Francesca Fiori, Simone Battaglia, Vincenzo Romei, Alessio Avenanti

**Affiliations:** 1 Centro Studi e Ricerche in Neuroscienze Cognitive, Dipartimento di Psicologia “Renzo Canestriari”, Campus di Cesena, Alma Mater Studiorum Università di Bologna, 47521 Cesena, Italy; 2 Precision Neuroscience & Neuromodulation Program, Gordon Center for Medical Imaging, Massachusetts General Hospital & Harvard Medical School, Boston, MA 02114, USA; 3 Dipartimento di Psicologia, Sapienza Università di Roma, 00185 Rome, Italy; 4 Institut für Klinische und Gesundheitspsychologie, Universität Wien, 1010 Vienna, Austria; 5 NeXT: Unità di Ricerca di Neurofisiologia e Neuroingegneria dell’Interazione Uomo-Tecnologia, Dipartimento di Medicina, Università Campus Bio-Medico, 00128 Rome, Italy; 6 Centro de Investigación en Neuropsicología y Neurociencias Cognitivas, Universidad Católica del Maule, Talca 346000, Chile

**Keywords:** aging, connectivity, plasticity, premotor cortex, motor cortex, motor performance, transcranial magnetic stimulation

## Abstract

Aging is commonly associated with a decline in motor control and neural plasticity. Tuning cortico–cortical interactions between premotor and motor areas is essential for controlling fine manual movements. However, whether plasticity in premotor–motor circuits predicts hand motor abilities in young and elderly humans remains unclear. Here, we administered transcranial magnetic stimulation (TMS) over the ventral premotor cortex (PMv) and primary motor cortex (M1) using the cortico–cortical paired-associative stimulation (ccPAS) protocol to manipulate the strength of PMv-to-M1 connectivity in 14 young and 14 elderly healthy adults. We assessed changes in motor-evoked potentials (MEPs) during ccPAS as an index of PMv-M1 network plasticity. We tested whether the magnitude of MEP changes might predict interindividual differences in performance in two motor tasks that rely on premotor-motor circuits, i.e., the nine-hole pegboard test and a choice reaction task. Results show lower motor performance and decreased PMv-M1 network plasticity in elderly adults. Critically, the slope of MEP changes during ccPAS accurately predicted performance at the two tasks across age groups, with larger slopes (i.e., MEP increase) predicting better motor performance at baseline in both young and elderly participants. These findings suggest that physiological indices of PMv-M1 plasticity could provide a neurophysiological marker of fine motor control across age-groups.

## 1. Introduction

Aging is commonly described as progressive physiological changes in an organism that lead to senescence and a decline in a variety of cognitive and biological functions [1,2,3,4,5,6]. In neuroscience, aging is usually associated with a progressive decrease in motor abilities, including a deterioration of fine motor control. Even when healthy, aging is accompanied by a continuing dwindling in motor functions that are essential to everyday living, such as manual dexterity and object manipulation [7,8]. This decline can be ascribed to several causes, including age-related modifications of the central nervous system [9,10] and the reported brain-wide changes at the structural and functional level observed in old age [2]. Concerning the sensorimotor networks, gray matter atrophy is reported in the precentral and postcentral gyri [3,4]; furthermore, older adults show reduced white matter volume and density relative to younger adults [5,6], and other structural and functional alterations over sensorimotor areas, that correlate with poor motor performance [3,4,5,10,11,12,13,14]. 

Neurophysiological studies have also documented altered cortico–cortical connectivity between premotor areas and the primary motor cortex (M1) in aging [15,16,17,18]. For example, studies have used transcranial magnetic stimulation (TMS) to investigate the strength of connectivity between the supplementary motor area (SMA) and M1, and reported that the conditioning effect exerted by SMA stimulation over M1 excitability is reduced in older adults compared to younger counterparts, indexing weaker SMA-to-M1 connectivity [16,17]; moreover, greater modulatory effect of SMA conditioning over M1 was associated with better motor performance, suggesting that the efficiency of SMA-to-M1 projections predicted individual differences in motor abilities [16]. 

The capability of a brain network to adapt to experience—i.e., the plasticity of the network—is a main feature of its efficiency. According to the Hebbian principle, interactions between neurons are dynamically shaped based on spiking activity: synapses are potentiated when presynaptic neurons repeatedly and coherently fire immediately before postsynaptic neurons. This concept is broadly referred to as spike-timing-dependent plasticity (STDP) [19,20,21,22]. Growing evidence suggests that plasticity is altered in the aging brain and, more specifically, animal studies found a reduction in STDP with advanced age [23,24,25,26,27]. However, to date, evidence that age-related modifications of cortical plasticity in humans predict reduced behavioral performance is still meager [28,29,30]. 

A valuable protocol for studying brain plasticity at the network level is the cortico–cortical paired associative stimulation (ccPAS) TMS paradigm. The ccPAS protocol is a dual-coil TMS method for inducing Hebbian associative plasticity between targeted brain areas. It consists of the repeated application of pairs of TMS pulses over two cortical areas [31,32,33,34,35,36]; in each pair, the pulse over the first stimulated target node (containing the “pre-synaptic neurons”) is immediately followed by a second pulse over a connected node (containing the “post-synaptic neurons”) with an optimal inter-stimulus interval (ISI) so as to mimic a pattern of neuronal stimulation ideal for inducing STDP.

A series of studies have successfully applied ccPAS in the motor system [34,35,37,38,39,40,41,42], particularly over the PMv-M1 network, showing effective modulation of motor excitability [43,44,45,46,47,48] and hand motor functions [46,49]. However, to date, these results have been mainly observed in young adults. One study applied a single dose of ccPAS over the posterior parietal cortex (PPC) and M1 in Alzheimer’s disease patients and healthy elderly controls and found ccPAS to induce a MEP increase only in the latter group [50], in line with neurophysiological evidence of preserved PPC-M1 connectivity in healthy elderly individuals but not Alzheimer’s disease patients [51]. On the other hand, to the best of our knowledge, only one study conducted in our lab has applied ccPAS over the PMv-M1 circuit in older individuals [49]: we administered PMv-M1 ccPAS in young and elderly participants, and found that while the protocol induced neurophysiological and behavioral effects coherent with the principles of STDP in young individuals, it did not have the same effect in the elderly group [49]. Our findings indicate that enhancing PMv-to-M1 connectivity via ccPAS consistently improved fine manual performance in young adults, moreso than in the elderly group, thus indicating a different effectiveness of the ccPAS protocol in the two age cohorts. Furthermore, while young participants displayed a progressive MEP increase during the ccPAS administration [46,48], elderly individuals did not consistently show this modulation [49]. These findings appear in line with the above mentioned evidence of altered premotor–motor connectivity in healthy elderly individuals [15,16,17,18]. 

However, while cortico–cortical plasticity of a network reflects a key feature of its efficacy, a relevant and so far unanswered question is whether age-related modifications of PMv-M1 plasticity in humans are associated with reduced behavioral performance. To fill this gap, we leveraged our recent ccPAS study [49] to investigate the relation between physiological changes induced by ccPAS and baseline manual motor performance across healthy elderly and young individuals. The ccPAS parameters we decided to adopt (i.e., the intensities of PMv and M1 stimulations and the interstimulus interval between them in each paired stimulation) were selected to repeatedly activate and strengthen a facilitatory cortico–cortical pathway from PMv to M1; indeed, based on studies conducted in our lab, the ccPAS protocol used here recruits facilitatory PMv-to-M1 connections [47]: that is, on each paired PMv-M1 stimulation, PMv conditioning would affect excitatory interneurons in M1, which in turn would be targeted by the second pulse over M1, influencing corticospinal neurons [46,47,48]. The repeated targeting of such facilitatory PMv-to-M1 circuit was found to induce a gradual MEP increase during ccPAS administration in the overwhelming majority of healthy young participants [48]. 

Specifically, we monitored the gradual MEP increase observed during ccPAS [47,48,49], reflecting the cortico–cortical plasticity of the PMv-M1 network as a proxy of the network’s efficiency. If such a neurophysiological marker of Hebbian plasticity is an effective indicator of the functionality of the network, we expect that across age groups, MEP facilitation during ccPAS would predict interindividual differences in motor performance.

## 2. Materials and Methods

### 2.1. Participants

We tested 28 individuals, divided into 14 young adults and 14 elderly adults (see Table 1 for demographic details). All participants were right-handed, based on the Edinburgh Handedness Inventory [52] (mean score 88.5 ± 20.8), had normal or corrected-to-normal vision, naïve to the purpose of the experiment and had no contraindication to TMS [53]. According to the Mini Mental State Examination (MMSE, mean corrected score 27.3 ± 2.1, range 24.2–28.4) and the Raven’s 100 colored progressive matrices (mean corrected score 29.8 ± 4.8, range 29–39), older individuals were not affected by age-associated cognitive deficits. Furthermore, they showed adequate power and precision grip strength, as measured by a force transducer, necessary to the execution of the selected visuomotor tasks. 

All the experimental procedures were performed in accordance with the 1964 Declaration of Helsinki and later amendments [54], and approved by the Department of Psychology “Renzo Canestrari” Ethical Committee and the Bioethics Committee at the University of Bologna. During the experiment, the recommended safety procedure for non-invasive brain stimulation administration during the COVID-19 pandemic was followed [55]. No adverse reactions or TMS-related discomfort were reported by participants or noticed by the experimenters.

### 2.2. Behavioral Tasks

To evaluate baseline motor performance, participants were asked to execute the 9-Hole Peg Test (9HPT), which assesses fine manual dexterity, and a choice reaction task (cRT) to assess the speed of visuomotor transformation. The 9HPT is a test commonly used to evaluate fine manual dexterity, as it requires participants to finely adjust and shape their hand to manipulate small objects (i.e., the pegs) to place them one by one into small holes [56,57]. The 9HPT apparatus consisted of a plastic board with 9 small holes organized in a three-by-three matrix. The distance between the holes was 3.2 cm, and pegs were placed in a tray of 8.5 × 10.4 × 2.3 cm fixed adjacent to the board. After receiving the start command, participants were instructed to press the space bar on a keyboard placed close by to start a clock; then, they had to pick up the nine small pegs with their right hand and put them one by one into one of the nine holes, and subsequently remove them one by one, returning them to the box; finally, they pressed the same space bar to stop the clock and record the performance speed of each trial. Participants were instructed to execute the task as quickly as possible. 

To assess alertness and manual speed, we employed a cRT; in particular, we used a 2-choice version of the cRT. In this version of the task the participants had to respond by releasing the key pressed by the index or middle finger of the right hand according to the number ‘1′ or ‘2′ displayed with equal probability on a monitor placed ~80 cm in front of them. Participants were instructed to perform the task as quickly and accurately as possible. Task accuracy (% of correct response) and mean reaction times (RTs) of correct responses were collected for each session.

Evidence indicates that performance in 9HPT and cRT is associated with activation of sensorimotor areas, including PMv and M1 [58], and brain stimulation over these regions was found to modulate performance of both these tasks [46,49,59,60,61,62]. 

After a brief training phase (~10 min), participants were asked to perform the two tasks in two separate blocks. In each of the two blocks, participants performed 5 iterations of the 9HPT and 40 trials of the cRT. Data from these two blocks were averaged. Motor performance was also tested in two blocks after ccPAS; results on the aftereffects of ccPAS in both groups have been reported elsewhere [49]. In the presented research, we focused on the relation between neurophysiological indices of brain plasticity during ccPAS (see below) and individual differences in motor performance (9HPT and cRT) as measured before ccPAS. 

### 2.3. ccPAS Procedure and Electrophysiological Recordings 

**Figure 1:**
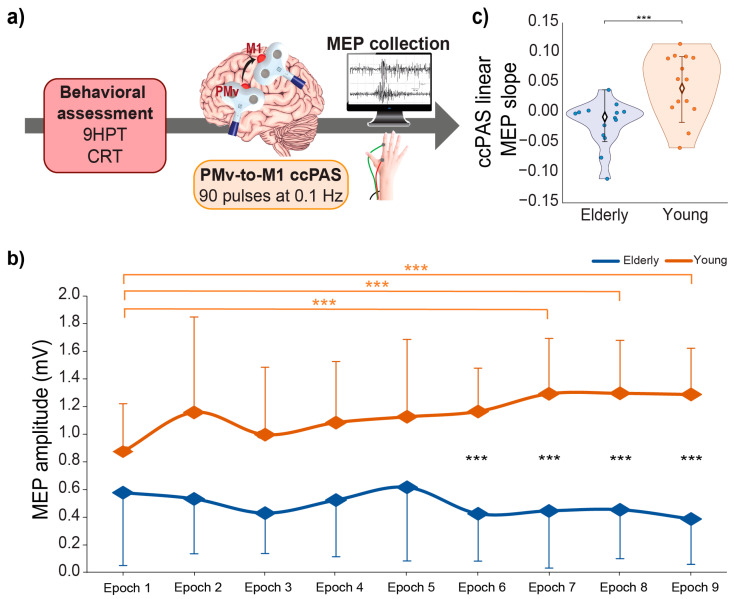
(**a**) Experimental design. Behavioral assessment was followed by the administration of a ccPAS protocol over the left PMv and M1. For each paired PMv-M1 stimulation of the ccPAS protocol, an MEP was collected from the right FDI; (**b**) mean MEP amplitudes recorded during the ccPAS in elderly (blue) and young (orange) participants along 9 epochs; (**c**) linear slope of MEP increase during ccPAS in the two groups. Error bars represent standard deviations; *** *p* ≤ 0.001.

ccPAS was administrated over the left PMv-to-M1 circuit in all participants. We set TMS intensity and coil positions before the ccPAS protocol, which consisted of 15 min of dual-site TMS delivered at a rate of 0.1 Hz (90 pairs of pulses; Figure 1a). In each pair, PMv stimulation preceded M1 stimulation by 8 ms to best activate the PMv-to-M1 pathway [47,63]. Indeed, while PMv-M1 cortico–cortical interactions occur at different time scales [63,64,65], the most consistent interstimulus interval (ISI) to condition M1 activity with PMv stimulation in an early window is a 8 ms ISI [47,63].

The PMv pulse intensity was set at 90% of the individual’s resting motor threshold (rMT), defined as the minimum stimulator output intensity necessary to induce MEPs ≥ 50 μV in 5 out of 10 consecutive trials [66] in the relaxed first dorsal interosseous (FDI). The intensity of the M1 pulse was adjusted to evoke MEPs with an amplitude of ~1 mV [39,43,45,46,48]. Using dual-coil TMS we have previously used the same stimulation parameters and found that subthreshold PMv stimulation administered 8 ms before suprathreshold M1 stimulation is optimal to target PMv-to-M1 excitatory interactions [47]; moreover, using the same PMv-to-M1 ccPAS protocol, we have reported lasting increases of motor excitability and reduction of GABA-ergic intracortical inhibition [47], that are preceded by a progressive MEP increase already during protocol administration [46,48].

Pulses delivered during the ccPAS were triggered remotely using a custom MATLAB script (MathWorks, Natick, MA, USA). To minimize discomfort, before starting the ccPAS we made participants experience PMv stimulation, using 3–4 pulses of increasing intensity. The stimulation was well tolerated by all participants.

The coil position to target the left M1 was identified functionally as the hotspot to induce MEPs of maximal amplitude in the relaxed right FDI. The left PMv was identified using the SofTaxic Navigator System (Electro Medical System, Bologna, Italy) as the scalp region overlying the Talairach coordinates: x = −52; y = 10; z = 24 [46,48]. These coordinates were determined by averaging previously reported coordinates [67,68,69,70,71]; these studies showed that stimulating this ventral frontal site (at the border between the anterior sector of the PMv and the posterior sector of the inferior frontal gyrus) affected planning, execution and perception of hand actions [72,73]. In all participants, skull landmarks (nasion, inion and 2 preauricular points) and ~80 points providing a uniform representation of the scalp were digitized by means of a Polaris Vicra digitizer (Northern Digital, Ontario, Canada). An individual estimated magnetic resonance image (MRI) was obtained for each participant through a 3D warping procedure fitting a high-resolution MRI template to the participant’s scalp model and craniometric points. The Talairach coordinates corresponding to the projections of the left PMv and left M1 scalp sites onto the brain surface were automatically estimated by the SofTaxic Navigator from the MRI-constructed stereotaxic template. No significant differences were found between the resulting Talairach coordinates in the two age groups (Table 2). 

Coils were held to induce current flows consistent with previous dual-site TMS and ccPAS studies targeting PMv and M1 [43,63,74]: the left PMv coil was placed tangentially to the scalp, inducing a current pointing toward the left M1; the left M1 coil was placed tangentially to the scalp and oriented at a ~45 angle to the midline, inducing a posterior-to-anterior current flow, optimal for M1 stimulation [75].

### 2.4. Electrophysiological Recording 

Because M1 stimulation during ccPAS was set at a suprathreshold intensity, we were able to record a MEP elicited by each of the 90 paired stimulations, thus allowing us to monitor online changes in corticomotor excitability [46,47,48,49] (Figure 1b). MEPs were recorded from the right FDI by means of surface Ag/AgCl electrodes placed in a belly–tendon montage. A Biopac MP-35 (Biopac, Goleta, CA, USA) electromyograph was used to acquire EMG signals (band-pass filter: 30–500 Hz; sampling rate: 20 kHz).

### 2.5. Data Analyses 

**Figure 2:**
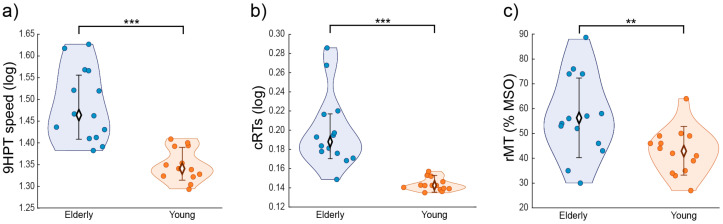
(**a**) 9HPT performance; (**b**) cRTs; and (**c**) rMT in young (orange) and elderly (blue) individuals. Error bars represent standard deviations; ** *p* ≤ 0.01; *** *p* ≤ 0.001.

MEP amplitudes, rMTs and the coordinates of the targeted brain sites were all normally distributed according to visual inspection and Kolmogorov–Smirnov tests (all *p* ≥ 0.20); to address normality violations, cRT and 9HPT values (both expressed in seconds) were log-transformed [log(value + 1)]. Then, parametric independent t-tests were used to compare age (Table 1), coordinates of the targeted brain sites (Table 2), log-transformed 9HTP and cRT values and the rMT (Figure 2c) between the two groups, while a non-parametric χ^2^ test with Yate’s correction was adopted to compare gender differences (Table 1). MEPs during ccPAS were assessed by measuring peak-to-peak EMG amplitude (in mV); MEPs ≤ 50 µV or preceded in the 100 ms before the pulse by EMG activity deviating ≥ 2 standard deviations from the subject’s rectified mean were discarded (11% of total). MEPs were grouped into 9 epochs of 10 trials each and averaged. Mean MEPs were analyzed with an analysis of variance (ANOVA) with the between-subjects factor age group (2 levels: young, elderly) and the within-subjects factors epoch (9 levels). Significant interactions were explored through Tukey’s post-hoc tests. As an index of individual modulation of corticomotor excitability during the ccPAS protocol, the linear slope of mean MEPs across the 9 epochs was computed for each participant (ccPAS linear MEP slope). To investigate whether neurophysiological indices of Hebbian plasticity predicted baseline motor performance we performed two general regression models, testing the efficacy of MEP increase during ccPAS (i.e., the ccPAS linear MEP slope) and its interaction with the age group (two levels: young and elderly) as predictors of baseline motor performance (9HPT performance speed and cRTs).

## 3. Results

The analysis showed a significant difference in baseline performance between the groups in both motor tasks. In particular, younger participants showed better motor performance than elderly participants, indexed by faster log-transformed execution times in the 9HPT (*t*_26_ = 5.66, *p* < 0.001; Figure 2a), which measures manual dexterity (raw 9HPT values, young: 21 ± 2 s; older: 30 ± 6 s), and by faster log-transformed RTs in the cRT task (*t*_26_ = 5.35, *p* < 0.001; Figure 2b), which measures alertness and visuomotor speed (raw cRT values, young: 391 ± 23 ms; older: 587 ± 150 ms). Additionally, baseline corticospinal excitability was significantly different between the two groups, as elderly individuals had a higher rMT compared to their younger counterparts (young: 43 ± 9%; older: 57 ± 16% of maximal stimulator output; *t*_26_ = 2.80, *p* = 0.009, Figure 2c). Critically, we found differences in the modulation of corticomotor excitability in young and older adults: the ANOVA on epoched MEPs recorded during the ccPAS revealed a main effect of the age group (*F*_1,26_ = 24.83, *p <* 0.001, *η_p_*^2^ = 0.49), qualified by a significant age group x epoch interaction (*F*_8,208_ = 3.63, *p* < 0.001, *η_p_*^2^ = 0.12). MEPs recorded during the protocol gradually increased in young participants showing significantly larger amplitudes in Epochs 7–9 with respect to Epoch 1 (all *p* ≤ 0.006; see Figure 1b), while no consistent MEP modulation was observed in elderly participants (all *p* ≥ 0.73). Moreover, MEPs recorded in the two groups differed significantly starting from Epoch 6 (all *p* ≤ 0.004, Figure 1b). Coherently, the ccPAS linear MEP slope recovered by fitting the 9 epochs to a linear model differed between the two groups, with young participants having a greater slope relative to elderly participants (*t*_26_ = −3.62, *p* = 0.001, Figure 1c). While the ccPAS linear MEP slope differed from zero in the young group (*t*_13_ = 3.22, *p* = 0.007), it did not in the elderly sample (*t*_13_ = −1.73, *p* = 0.11).

**Figure 3:**
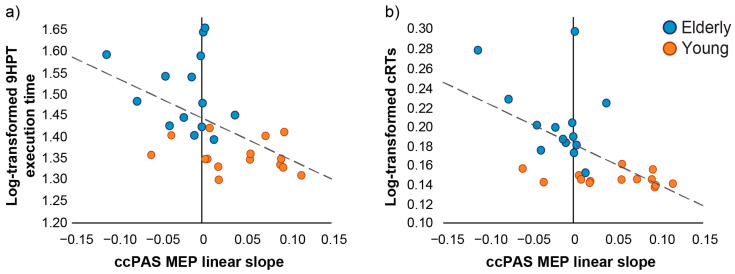
Relation between the neurophysiological marker of STDP (ccPAS MEP linear slope) and motor performance assessed at baseline. The STDP index predicts both 9HPT execution times (**a**) and cRTs (**b**) execution times across age groups, showing that larger MEP slope (reflecting greater plasticity) is associated with faster motor performance at baseline. Orange dots represent young participants (N = 14) and blue dots represent elderly participants (N = 14). Dashed lines depict the regression line of the significant predictor ccPAS MEP linear slope on 9HPT execution times (**a**) and cRTs (**b**) across groups.

The regression models between the linear slope of the MEP modulation induced by ccPAS (ccPAS linear MEP slope) and baseline motor performance were significant (9HPT: *R*^2^*_adj_* = 0.22, *F*_2,25_ = 4.71, *p* = 0.02; cRTs: *R*^2^*_adj_* = 0.30, *F*_2,25_ = 6.73, *p* = 0.005, Figure 3), with individual differences in MEP slope predicting individual differences in motor performance (9HPT: *β* = −0.53, *p* = 0.009; cRTs: *β* = −0.64, *p* = 0.001). The negative relationship between MEP slope and motor performance at baseline indicates that individuals who showed greater physiological sensitivity to ccPAS manipulation also exhibited faster execution times in both tasks. This effect was similar across groups: indeed, the interaction with the predictor age group was not significant in either regression model (9HPT: *β* = −0.01, *p* = 0.94; cRTs: *β* = −0.18, *p* = 0.32). Thus, these results indicate that the ccPAS linear MEP slope similarly predicted individual differences in motor performance across age groups. Moreover, partial correlations showed that the association between MEP slope and motor performance across groups remained significant (9HPT: −0.53, *t*_25_ = −3.10, *p* = 0.005; cRTs: −0.57, *t*_25_ = −3.47, *p* = 0.002) even when controlling for the influence of corticomotor excitability (i.e., rMT) (9HPT: 0.33, *t*_25_ = 1.77, *p* = 0.09; cRTs: 0.27, *t*_25_ = 1.40, *p* = 0.17). Taken together, the results of the regression models and partial correlations indicate that the ability of PMv-M1 ccPAS to enhance corticomotor excitability predicts baseline hand motor dexterity and speed performance. This measure represents a key neurophysiological marker of the preserved plastic properties of the PMv-M1 circuit and can serve as a proxy for the motor functions supported by this network.

## 4. Discussion

Neural plasticity underlies the capability of the brain to adapt its structure and function in response to experience. This capacity is fundamental, as it allows one to cope with changes in the internal and external environment long after infancy [1,76]. However, the aging process can undermine the plastic properties of the brain across different networks, including the motor system [1,18,28,29,30]. It has been argued that a healthy brain is a changing brain [77,78] and, accordingly, that efficient and flexible cortico-cortical networks should be characterized by neural plasticity. Non-invasive brain stimulation techniques, such as TMS-paired associative stimulation protocols, have been proposed as a method to track plasticity across the lifespan, and thus have been regarded as method to index brain health [77,78]. Using ccPAS to provoke mechanisms of Hebbian plasticity over the PMv-to-M1 pathway, we have previously demonstrated that cortico–cortical plasticity of this pathway decreases with aging [49]. Indeed, studies conducted in our lab found that young participants showed increased 9HTP performance following ccPAS, thus supporting the critical role of the PMv-M1 network in visually guided fine motor control [79,80] and confirming that PMv-M1 ccPAS can enhance this sensorimotor function [46]; in contrast, elderly individuals did not exhibit an increase at a group level [49]. The application of the ccPAS protocol with M1 suprathreshold stimulation allowed us to track corticomotor excitability during the entire ccPAS intervention and derive an index of the plastic response of the targeted network; while we observed a linear increase of MEPs in young adults during ccPAS administration [46,48], no similar change was found in older adults in the present (Figure 1) and previous study [49]. We interpret this linear increase in motor excitability observed during PMv-M1 ccPAS as a result of the progressive increase of PMv-M1 interaction efficacy [46,47,48] and the expression and build-up of Hebbian plasticity due to the repeated and coherent activation of an excitatory PMv-to-M1 pathway [39,41,42,43,44,45,46,47,48]. Interestingly, these changes can be of variable size in young and older individuals [46,49], possibly reflecting individual differences in the plastic potential of the PMv-M1 pathway. 

A decrease in manual dexterity is commonly observed in older adults and, although this can be partially ascribed to peripheral changes affecting muscles or nerves, evidence of reduced white matter volume and density in the elderly sensorimotor system [3,5,6] hints at the contribution of impaired cortico-cortical connectivity to age-related reductions in motor control efficiency [15,16,17,18]. Hence, in this study, we hypothesized that age-related differences in fine manual control might reflect the efficiency of the PMv-M1 network, which is crucial for transforming sensory stimuli into appropriate motor commands during manual performance [46,79,80]. As a neurophysiological index of PMv-M1 network efficiency, we evaluated the linear increase of corticomotor excitability during PMv-M1 ccPAS administration, i.e., the ccPAS MEP linear slope, which reflects the plasticity of the targeted network. We tested whether this neurophysiological index would predict age-related individual differences in fine manual control. We assessed motor performance at baseline, before any ccPAS intervention, using two established motor tasks, namely the 9HPT and cRT, which are used to evaluate hand motor dexterity and visuomotor speed and have been associated with activation of premotor–motor areas [6,58,81,82,83]. As reported previously [49], these results confirm prior findings of decreased manual motor performance in the elderly, with slower 9HPT and cRT performance [8,84]. Moreover, while young adults show sensitivity to ccPAS administration, improving their performance after it, elderly participants present no modulation at a group level, suggesting that, on average, advanced age impairs the susceptibility to plastic changes in the PMv-M1 network [28,29,30]. 

The main goal and novel finding of the present research revolves around the relation between the increase of corticomotor excitability during ccPAS administration—reflecting an index of PMv-M1 plasticity and integrity—and individual differences in motor performance. As we predicted, across age groups, we observed a significant relation between the magnitude of MEP increase during ccPAS administration (ccPAS MEP linear slope) and baseline motor performance, suggesting that greater corticomotor modulations predicted better performance. In a similar vein, reduced corticomotor modulations predicted poorer performance in the two age groups. These findings suggest that greater plasticity reflects a more efficient and preserved PMv-M1 network, which would grant a better motor performance, whereas decreased plasticity of the targeted PMv-M1 network potentially underlies reduced functional efficiency.

These results held true even when controlling for baseline motor excitability (i.e., rMT values). Previous studies conducted in our lab found that baseline rMT values correlated with the extent of corticomotor excitability increase induced during the ccPAS and behavioral improvements [46,48]; our control analyses allow us to rule out the possibility that our findings are merely due to differences in rMT, rather than differences in PMv-M1 network plasticity and efficiency between young and older individuals.

The present findings significantly expand our previous results: here, we found that MEP increase during ccPAS predicts baseline motor abilities per se, not only their responsiveness to ccPAS manipulation. Notably, we observed this predictive efficacy both when using the 9HPT, which is the optimal task to tap into the functional output of the PMv-M1 network [58,59,61], and the cRT, which recruits the PMv to a lesser extent [46]. It is possible that reduced PMv-M1 plasticity is embedded in a generalized plasticity reduction that could affect the frontal nodes of the motor system. Thus, reduced PMv-M1 plasticity would reasonably correlate with poorer performance in a multitude of motor tasks. In this view, one of the critical limitations of the present study is the relatively few tasks we adopted, only testing fine dexterity via the 9HPT task and visuomotor reaction times via the cRT. Expanding to other domains, both within and outside of the motor system, would enrich our understanding of the relationship between plasticity and cognition. Moreover, we believe that future studies should address the topic of lifelong modifications in cortico-cortical plasticity, and their impact on behavior. Indeed, in our study we focused on two distinct groups of young (~23 years of age) and elderly healthy adults (~71 years of age); however, healthy brain aging is a lifelong gradual process [1] and, thus, further research including intermediate samples would yield relevant insights into the progression of plasticity changes into old age. 

Our sample included both female and male individuals of fertile and non-fertile age, with no statistical differences between age groups. However, our sample was not perfectly matched for gender and we did not assess ovarian hormones that could in principle affect sensitivity to TMS [85]. It should be noted, however, that prior work on classical PAS aftereffects would suggest little or no influence of gender [86]. Similarly, a study conducted in our lab on a substantial sample size (N = 109) found no appreciable differences between male and female participants in their responsiveness to a ccPAS protocol identical to the one adopted in the present study [48].

In conclusion, our results reveal that maintained physiological indices of STDP mechanisms seem to be an effective neurophysiological marker of health in premotor–motor chains not only in young adults but, critically, in the elderly as well. The extent of the corticospinal excitability modulation induced by ccPAS was found to predict baseline visuomotor performance in both our age groups; this indicates that synaptic plasticity could be considered a relevant index of the health and maintained efficiency of brain circuits. However, these findings also indicate that neuronal plasticity tends to physiologically reduce with age, which might negatively impact the feasibility and effectiveness of non-invasive brain stimulation techniques such as ccPAS. This raises the challenging question of how to determine the residual plastic potential of the aging brain and how to preserve and promote its network plasticity. This concern calls for further research into the implementation of non-invasive brain stimulation protocols to effectively induce associative plasticity in the healthy elderly population. Indeed, the study here presented adopted a well-established and replicated ccPAS protocol [39,43,46,47,48], which is informed by the PMv-M1 connectivity patterns and timings explored in healthy young adults [63,87], to repeatedly activate the targeted pathway in a way that is consistent with the Hebbian principle. Nonetheless, previous results indicate that the aging process can affect connectivity between the M1 and other premotor regions, such as the supplementary motor area [16,17]. Although there is currently no research specifically focusing on the PMv-M1 circuit, it is reasonable to assume that the motor systems of elderly adults may be characterized by altered cortico–cortical interactions. Therefore, investigating the implementation of protocols tailored to accommodate such physiological shifts would be recommended for future research.

Finally, the results of the present study yield insights into age-associated brain changes in the motor cortical neurocircuitry and the mechanisms underlying fine motor abilities across age groups.

## Figures and Tables

**Table 1:** Demographic information across the group groups.

Group	Age	Gender
Elderly	71.21 years ± 6.95	Males = 11, Females = 3
Young	23.08 years ± 2.91	Males = 6, Females = 8
Statistical analyses	*t*_26_ = 23.13, *p* < 0.0001	Yates’s *χ*^2^ = 2.40, *p* = 0.12

The table shows the mean age ± standard deviation, the number of males and females in the two age groups and the respective statistical comparisons.

**Table 2:** Mean Talairach coordinates of the stimulation sites in the two age groups.

Group	M1			PMv		
	x	y	z	x	y	z
Older	−33.6 ± 6.3	−18.6 ± 7.7	59.7 ± 4.2	−53.6 ± 2.0	9.6 ± 1.5	23.7 ± 1.1
Young	−30.5 ± 5.7	−16.5 ± 6.1	59.0 ± 4.8	−51.6 ± 2.2	9.1 ± 1.8	23.2 ± 2.6
Statistical analyses	No effect of group. All *t* ≤ 1.43, all *p* ≥ 0.14					

The table shows the mean Talairach coordinates ± standard deviations of the two target sites in young and older individuals.

## Data Availability

The raw data supporting the conclusions of this article will be made available by the authors without undue reservation.
